# Predictors of Flares in Patients with Rheumatoid Arthritis Who Exhibit Low Disease Activity: A Nationwide Cohort Study

**DOI:** 10.3390/jcm9103219

**Published:** 2020-10-07

**Authors:** Yoon-Jeong Oh, Ki Won Moon

**Affiliations:** Division of Rheumatology, Department of Internal Medicine, Kangwon National University School of Medicine, Chuncheon 24289, Korea; yjgark640@gmail.com

**Keywords:** rheumatoid arthritis, flare, prediction

## Abstract

Using nationwide cohort data, this study evaluated predictors of flares in patients with rheumatoid arthritis (RA) who exhibit low disease activity (LDA) and the effects of flares on clinical outcomes. The Korean Observational Study Network for Arthritis (KORONA) registry is a nationwide Korean RA-specific cohort registry that collects data annually from 5.077 patients, with RA in 23 centers across South Korea. This study used data from 1.717 patients with RA who exhibited LDA [28–joint disease activity score (DAS28) < 3.2] at enrollment. Flares were defined as an increase in DAS28, compared with the previous value of > 1.2 or > 0.6, if the concurrent DAS28 was ≥ 3.2. Cox regression analysis was used to identify baseline predictors of flares. Of the 1.717 patients with RA, 566 (33.0%) experienced flares during the 2-year study period. An analysis of baseline characteristics of flare and non-flare groups revealed that more women and non-smokers were present in the flare group than in the non-flare group; the flare group also had higher scores on physician’s and patient’s pain and fatigue visual analogue scales (VAS) and the health assessment questionnaire (HAQ). In a multivariate analysis, physician’s VAS score, hemoglobin level, and HAQ score were significant predictors of flares. A high physician’s VAS score, low hemoglobin, and high HAQ score at baseline were significant predictors of flares in patients with RA who exhibited LDA.

## 1. Introduction

The treatment of rheumatoid arthritis (RA) should target remission or low disease activity (LDA) in every patient [1]. If remission or LDA is reached, this state should be maintained. However, some patients with RA who exhibit LDA experience flares in their disease; up to 30% of patients with RA experience flares [2]. RA flares are typically defined as enhanced joint pain, joint swelling, and elevated levels of acute phase reactants. There is evidence that flares contribute to the worsening of subjective symptoms, joint damage, and other long-term outcomes [3]. In contrast, some patients remain in remission or exhibit LDA. The ability to predict flares when patients reach remission or LDA could facilitate decisions regarding treatment maintenance or reduction. There have been many studies about prediction for remission, but few studies about prediction for flare in RA. Bechman et al. evaluated predictors for flare in 97 RA patients who tapered tumor necrosis inhibitors after they reached sustained LDA [4]. They found baseline DAS28 and mental health status were predictors for flare. The results of another study of 93 RA patients with remission showed that ultrasound might be a useful tool for flare prediction [5]. In a recent study of 152 RA patients with LDA, health assessment questionnaire (HAQ) was a significant predictor for flare [6]. However, they were relatively small-sized and short-term observation studies. It is necessary to evaluate predicting factors for flare with large scale cohort database for long term period. Therefore, using nationwide cohort data, this study investigated predictors of flares in patients with RA who exhibited established LDA and the effects of flares on clinical outcomes in those patients.

## 2. Materials and Methods

### 2.1. Study Subjects

The Korean Observational Study Network for Arthritis (KORONA) is a nationwide Korean RA-specific cohort based on a prospective protocol and standard, with defined data collection instruments and annual patient follow-up. The KORONA cohort was established in July 2009 and enrolled patients with RA from July 2009 to December 2012. Patients aged 18 years or older who met the 1987 American College of Rheumatology criteria for diagnosis of RA were recruited by rheumatologists in 23 centers. KORONA is the largest nation-wide multicenter cohort; the demographic distribution of patients by age and sex in this cohort is comparable to that of all patients with RA in Korea. Demographic data, clinical features, laboratory data, radiologic findings, health-related outcomes, treatment modality, resource utilization, and health behaviors were collected from enrolled patients. A total of 5.077 patients were enrolled at baseline and participated in annual follow-ups. The KORONA protocol was approved by the Institutional Review Board (IRB) of all participating hospitals, and all participants were provided informed consent prior to enrollment in the study. This study was approved by the IRB of Kangwon National University Hospital.

### 2.2. Clinical Assessment

Data were collected regarding demographic and socioeconomic factors (i.e., sex, age, body mass index (BMI), smoking, marital status, regular exercise, and monthly income), as well as clinical factors (i.e., disease duration, familial history, side effects, poor adherence, malignancy, operation history, fracture history, and number of combined disorders). Disease activity and functional disability were measured using a physician’s visual analogue scale (VAS), patient’s pain VAS, global health VAS, fatigue VAS, 28-joint count disease activity score (DAS28)-ESR, EuroQol-5D (EQ-5D), and HAQ. Laboratory data were collected regarding rheumatoid factor, anti-citrullinated protein antibody (ACPA), hemoglobin, erythrocyte sedimentation rate (ESR), and C-reactive protein (CRP); medication data were collected regarding methotrexate, disease-modifying anti-rheumatic drugs, nonsteroidal anti-inflammatory drugs, steroids, and biologics.

### 2.3. Definitions of LDA and RA Flares

**Figure 1:**
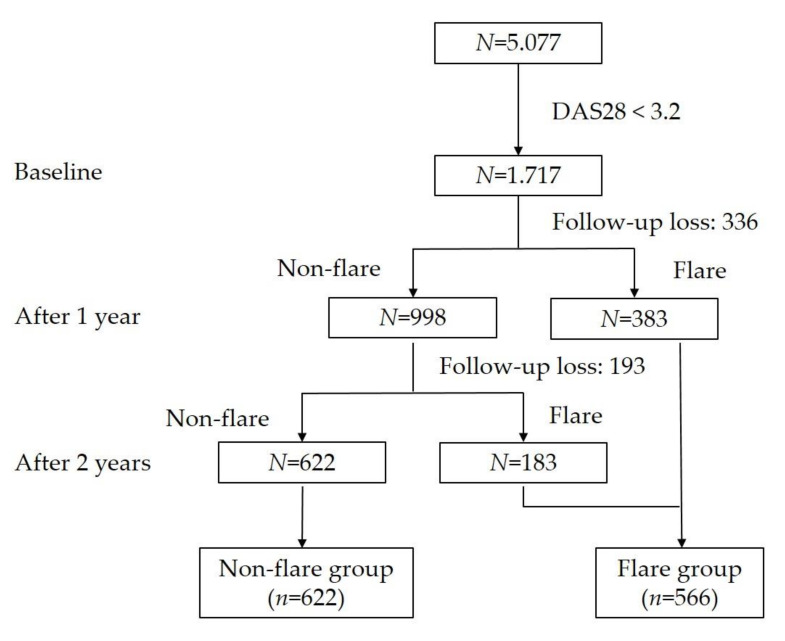
Flow chart of enrolled patients with rheumatoid arthritis during the 2-year study period.

We enrolled patients with RA who exhibited LDA and had a baseline DAS28-ESR of <3.2. Flares were defined using validated criteria as a DAS28 increase of >1.2, compared with the previous value, or a DAS28 increase of >0.6 with concurrent DAS28 of ≥3.2 [7]. We investigated flares in patients with RA who exhibited LDA for total 2 years. After 1 year, we separated patients with and without flares into flare and non-flare groups according to a reported definition; patients without flares at 1 year were divided into flare and non-flare groups after 2 years. We allocated patients who had not experienced a flare for 2 years into the non-flare group and patients who had experienced at least one flare into the flare group (Figure 1). We compared their clinical outcomes after 2 years, including changes in physician’s VAS score, patient’s pain VAS score, global health VAS score, fatigue VAS score, DAS28-ESR, EQ-5D score, and HAQ score.

### 2.4. Statistical Analysis

Descriptive analysis was used to summarize the baseline characteristics of both groups. The chi-squared test and Student’s *t*-test were used to compare categorical and continuous variables, respectively. The multiple imputation method was used to input missing continuous values. To identify significant predictors of flares, univariate and multivariate Cox regression analyses were performed. These results were expressed as hazard ratios with 95% confidence intervals. Student’s *t*-test was used to compare changes in clinical outcomes between groups at 2 years. Results with *p*-values < 0.05 were considered statistically significant. All analyses were performed using IBM SPSS ver. 24.0 (IBM Corp., Armonk, NY, USA).

## 3. Results

From the 5.077 patients with RA, we selected 1.717 patients who had DAS28-ESR < 3.2 at baseline. After 1 year, 336 patients were lost to follow-up, 338 patients had a flare, and 998 patients remained in a non-flare state. After the 2-year follow-up, 193 patients were lost to follow-up, 183 patients had a flare, and 622 patients remained in a non-flare state. Consequently, 622 patients were allocated to the non-flare group and 566 patients were allocated to the flare group (Figure 1).

Table 1 shows the baseline characteristics of both groups. The proportions of women and non-smokers were higher in the flare group, while the proportion of past smokers was higher in the non-flare group. The physician’s VAS, patient’s pain VAS, and fatigue VAS scores were higher in the flare group. The EQ-5D score was higher in the non-flare group, while the HAQ score was higher in the flare group. The proportion of ACPA-positive patients and levels of hemoglobin were both higher in the non-flare group. The flare group tended to have a longer disease duration and greater incidence of side effects, although these differences were not statistically significant. We divided flare group into first year and second year flare group to find out differences in patients flaring after first or second year (Supplementary Table S1). Most of baseline characteristics were not significantly different, except the frequency of poor adherents and NSAID users. The frequency of poor adherents was higher in second year flare group, and that of NSAID users was higher in first year flare group.

Table 2 shows the results of Cox proportional hazards analyses. A univariate analysis revealed that female sex, past smoker status, physician’s VAS score, pain VAS score, fatigue VAS score, hemoglobin level, EQ-5D score, and HAQ score at baseline were significant predictors of flares. These variables were then entered into multivariate analysis. Flares were associated with enhancements of physician’s VAS and HAQ scores, as well as with the reduction of hemoglobin level. We reanalyzed the data from 687 RA patients who exhibit remission (DAS28 < 2.6) at baseline level, with univariate and multivariate Cox regression models for flare prediction (Supplementary Table S2). The results were similar with those of data from RA patient with LDA. Physician’s VAS, hemoglobin, and HAQ were still significant predictors for flare.

Table 3 shows the changes in clinical outcomes at 2 years in the non-flare and flare groups. The changes in physician’s VAS score, patient’s pain VAS score, global health VAS score, fatigue VAS score, DAS28, EQ-5D score, and HAQ score differed significantly between the two groups.

## 4. Discussion

This study investigated the predictors of flare in patients with RA who exhibited LDA for 2 years using data from a large cohort. Of 1.717 patients with RA, 566 (33.0%) experienced a flare at least once during the 2-year study period. The prevalence of flare differs according to the follow-up interval, observation period, and flare definition. Flares can be defined based on subjective symptom worsening, a questionnaire, or a disease activity score, such as the DAS28. By investigating flares using a questionnaire every 6 months, Bykerk et al. reported that 95% of patients experienced at least one flare, with a frequency of 54~74% per evaluation during a 3-year study period [2]. However, in another study where flare was defined based on the DAS28, 46 (30%) of 152 patients experienced a flare within the 1-year study period [6]. Flares seem to be more frequent when defined based on subjective symptoms, rather than objective disease activity. The Outcome Measures in Rheumatology (OMERACT) initiative classified the RA flare criteria based on changes in the DAS28 [7]; therefore, we applied the flare criteria suggested by OMERACT. Recently, the OMERACT initiative recognized the limitations of the DAS28 in defining flare events and suggested a consensus-based core domain set to identify flares in patients with RA [8].

The baseline characteristics flare and non-flare groups were significantly different in regard to sex, smoking, subjective symptom, functional status, and laboratory results. However, when compared to the baseline characteristics of first year and second year flare groups, the only differences were adherence and NSAID use. The percentage of NSAID users were higher in first year flare group, which would have artificially lowered the baseline DAS28, leading to flare in earlier time. Our results indicated that baseline physician’s VAS score, hemoglobin, and HAQ score predicted flares in patients with RA who exhibited LDA. Notably, the physician’s VAS score predicted flares in patients with RA, whereas the patient’s VAS score did not. Some studies have reported a discrepancy between patient’s VAS and physician’s VAS scores. Furu et al. reported that the inflammatory burden (e.g., swollen joint count, tender joint count, and CRP) mainly contributed to the physician’s VAS score, whereas non-inflammatory variables (e.g., pain and functional disability) influenced the patient’s VAS score [9]. The physician’s VAS score could predict flares, whereas the patient’s VAS score could not, because flares were defined based on DAS28-ESR in our study. The hemoglobin level is known to be negatively correlated with disease activity [10]. A low hemoglobin level has also been shown to predict radiographic progression in patients with RA. Möller et al. reported that a lower hemoglobin level was associated with greater radiographic damage, even in patients with LDA [11]. Furthermore, there have been reports that HAQ score is an independent predictor of flares in patients with RA. Bechman et al. investigated predictors of flares in 152 patients with RA who exhibited LDA; they found that a higher baseline HAQ score was a modest predictor of flares [6]. Another Korean study investigated potential predictors of sustained remission in 290 patients with RA from one center; the findings suggested that only the baseline HAQ score was independently associated with sustained remission (according to the DAS28-CRP) for 2 years [12]. Moreover, the HAQ score has been associated with mortality in patients with RA. Michaud et al. evaluated whether functional disability could predict mortality in 10.319 patients with RA from the USA; they found that HAQ and short-form 36 questionnaire scores were strongly associated with mortality risk in patients with RA [13]. Many other variables are known to predict flares in patients with RA. A study in the UK revealed that female sex, higher tender joint count, higher HAQ score, obesity, hypertension, and depression were independent predictors of flares [14]. Power Doppler activity has also been reported to predict flares. Saleem et al. showed that elevated baseline ultrasound power Doppler activity and functional disability were independently associated with the risk of flares [5]. Overall, the HAQ score appears to be an important predictor of flares, clinical outcomes, and radiographic progression.

We demonstrated that flares affected the clinical outcome in patients with RA who exhibited LDA for a relatively long duration. There were significant differences in clinical outcomes over 2 years between the non-flare and flare groups; these outcomes included changes in the physician’s VAS score, patient’s pain VAS score, global health VAS score, fatigue VAS score, EAS28, EQ-5D score, and HAQ score. Thus, flares contribute to worsening subjective symptoms, disease activity, quality of life, and functional capability; these results are consistent with the findings of other studies. One study evaluated the long-term effect of flares in 508 patients with RA (using data from The BeSt Study) over a period of 10 years. Greater numbers of flares were associated with greater disease activity, pain, morning stiffness, functional deterioration, and radiographic progression [3]. Another study observed 268 patients with RA who exhibited LDA for 2 years; the incidences of radiographic progression and functional impairment were higher in patients with flares than in patients without flares [15]. These results suggested that flares negatively affect clinical outcomes in patients with LDA, as well as patients with high disease activity.

There were some limitations in this study. First, we did not reflect a change in medication as a predictor of flares. However, our purpose was to identify significant predictors of flares at baseline. Second, we did not evaluate radiographic progression because the KORONA registry does not provide a quantitative measure of radiographic damage. Third, we could not monitor short-term flares, because a 1-year follow-up interval was used. However, we presume that our data provide an important perspective regarding the treatment of patients with RA who exhibit LDA. When patients with RA reach remission or LDA, many physicians consider reducing their medication in accordance with the management guidelines for RA [1]. However, it is necessary to individualize the treatment plans of patients who are expected to have flares, because flares are associated with symptom worsening, functional disability, and radiographic progression. Physicians should exercise caution when reducing the dose of medication if patients have a high physician’s VAS score, low hemoglobin level, and high HAQ score in conjunction with LDA.

## Figures and Tables

**Table 1:** Baseline characteristics of non-flare and flare groups.

	Non-Flare Group (*n* = 622)	Flare Group (*n* = 566)	*p*-Value
Female sex (%)	474 (76.2)	461 (81.4)	0.028 *
Age	52.55 ± 11.86	52.43 ± 12.11	0.862
BMI (kg/m^2^)	22.68 ± 3.06	22.64 ± 3.38	0.817
Socioeconomic data			
Smoking status (%)			
Non-smoker	470 (75.6)	469 (82.9)	
Past smoker	87 (14.0)	45 (8.0)	0.002 **
Current smoker	65 (10.5)	52 (9.2)	
Married (%)	507 (81.5)	462 (81.6)	1.000
Regular exercise (%)	314 (50.5)	273 (48.2)	0.450
Monthly income (USD, %)			
<2.000	377 (60.6)	359 (63.4)	
2.000–4.999	212 (34.1)	188 (33.2)	0.221
≥5.000	33 (5.3)	19 (3.4)	
Clinical data			
Disease duration (years)	5.94 ± 6.02	6.56 ± 6.25	0.082
Familial history (%)	86 (13.8)	73 (12.9)	0.670
Side effects (%)	179 (28.8)	192 (33.9)	0.060
Poor adherent (%)	25 (4.0)	30 (5.3)	0.334
Malignancy (%)	50 (8.0)	50 (8.8)	0.676
Operation history (%)	73 (11.7)	83 (14.7)	0.144
Fracture history (%)	96 (15.4)	89 (15.7)	0.936
Number of combined disorders	0.88 ± 1.09	0.88 ± 1.03	0.953
Physician’s VAS score (mm)	15.07 ± 12.90	18.73 ± 13.18	0.000 **
Pain VAS score (mm)	19.75 ± 20.63	22.28 ± 21.44	0.038 *
Global health VAS score (mm)	23.87 ± 20.55	24.80 ± 19.91	0.431
Fatigue VAS score (mm)	31.29 ± 26.25	35.14 ± 27.26	0.014 *
DAS28-ESR	2.32 ± 0.64	2.37 ± 0.63	0.299
EQ-5D score	0.82 ± 0.17	0.78 ± 0.17	0.001 **
HAQ score	0.27 ± 0.36	0.38 ± 0.45	0.000 **
Laboratory data			
RF (%)	310 (49.8)	279 (49.3)	0.862
ACPA (%)	447 (71.9)	372 (65.7)	0.024 *
Hemoglobin (g/dL)	13.02 ± 1.36	12.83 ± 1.26	0.015 *
ESR (mm/h)	14.26 ± 12.83	15.29 ± 12.28	0.157
CRP (mg/dL)	0.29 ± 1.01	0.31 ± 0.63	0.723
Medication data			
Methotrexate (%)	543 (87.3)	492 (86.9)	0.863
Number of DMARDs	1.76 ± 0.65	1.78 ± 0.65	0.625
NSAIDs (%)	483 (77.7)	431 (76.1)	0.581
Steroids (%)	426 (68.5)	382 (67.5)	0.756
Biologics (%)	37 (5.9)	28 (4.9)	0.523

ACPA, anti-citrullinated protein antibody; BMI, body mass index; CRP, C-reactive protein; DAS28, 28-joint count disease activity score; DMARD, disease modifying anti-rheumatic drug; ESR, erythrocyte sedimentation rate; EQ-5D, EuroQol-5D; HAQ, health assessment questionnaire; NSAID, non-steroidal anti-inflammatory drug; RF, rheumatoid factor; VAS, visual analogue scale; * *p* < 0.05; ** *p* < 0.01.

**Table 2:** Cox proportional hazards model of flares in patients with RA who exhibit low disease activity.

Variables	Univariate Analysis		Multivariate Analysis	
	HR (95% CI)	*p*-Value	HR (95% CI)	*p*-Value
Female sex	1.29 (1.04–1.59)	0.019 *	0.85 (0.60–1.21)	0.367
Smoking status				
Non-smoker	1 (reference)			
Past smoker	0.68 (0.50–0.93)	0.014 *	0.69 (0.46–1.03)	0.068
Current smoker	0.76 (0.57–1.01)	0.059	0.84 (0.57–1.22)	0.355
ACPA	0.86 (0.72–1.03)	0.095	0.92 (0.77–1.09)	0.324
Physician’s VAS score	1.02 (1.01–1.02)	0.000 **	1.01 (1.01–1.02)	0.000 **
Pain VAS score	1.01 (1.00–1.01)	0.012 *	0.99 (0.99–1.00)	0.506
Fatigue VAS score	1.01 (1.00–1.01)	0.012 *	1.01 (0.99–1.01)	0.146
Hemoglobin	0.90 (0.84–0.95)	0.001 **	0.93 (0.87–0.99)	0.047 *
EQ–5D score	0.46 (0.29–0.73)	0.001 **	0.90 (0.49–1.65)	0.732
HAQ score	1.55 (1.30–1.86)	0.000 **	1.41 (1.12–1.78)	0.003 **

Acpa, anti-citrullinated protein antibody; CI, confidence interval, EQ-5D, EuroQol-5D; HAQ, health assessment questionnaire; HR, hazard ratio; VAS, visual analogue scale; * *p* < 0.05; ** *p* < 0.01.

**Table 3:** Changes from baseline levels for 2-year clinical outcomes in non-flare and flare groups.

Clinical Outcome	Non-Flare Group	Flare Group	*p*-Value
Physician’s VAS score change	−4.40 ± 14.11	−1.18 ± 16.85	0.000 **
Pain VAS score change	0.83 ± 23.01	12.78 ± 28.77	0.000 **
Global health VAS score change	1.52 ± 22.72	15.20 ± 27.61	0.000 **
Fatigue VAS score change	−0.25 ± 31.35	7.81 ± 31.35	0.000 **
DAS28 change	−0.14 ± 0.71	1.07 ± 1.02	0.000 **
EQ-5D score change	0.01 ± 0.19	−0.05 ± 0.22	0.000 **
HAQ score change	0.00 ± 0.38	0.12 ± 0.49	0.000 **

DAS28, 28-joint count disease activity score; EQ-5D, EuroQol-5D; HAQ, health assessment questionnaire; VAS, visual analogue scale; ** *p* < 0.01.

## Supplementary Materials

The following are available online at https://www.mdpi.com/2077-0383/9/10/3219/s1, Table S1: Baseline characteristics of first year and second year flare groups, Table S2: Cox proportional hazards model of flares in patients with RA who exhibit remission.
