# Supplementary material for: Predictors of Flares in Patients with Rheumatoid Arthritis Who Exhibit Low Disease Activity: A Nationwide Cohort Study
Source: J Clin Med. 2020 Oct 7;9(10):3219. doi: 10.3390/jcm9103219 (PMC7601432; doi:10.3390/jcm9103219)
Supplement: Supplementary file 1 [file jcm-09-03219-s001.pdf]

Supplementary Table 1. Baseline characteristics of first year and second year flare groups.

	First year flare group (n=383)	Second year flare group (n=183)	*P*-value
Female sex (%)	311 (81.2)	150 (82.0)	0.908
Age	52.89±12.28	51.46±11.74	0.192
BMI (kg/m^2)	22.81±3.44	22.27±3.20	0.076
Socioeconomic data			
Smoking status (%)			
Nonsmoker	321 (83.8)	148 (80.9)	
Past smoker	28 (7.3)	17 (9.3)	0.650
Current smoker	34 (8.9)	18 (9.8)	
Married (%)	311 (81.2)	151 (82.5)	0.730
Regular exercise (%)	178 (46.5)	95 (51.9)	0.243
Monthly income (USD, %)			
<2,000	252 (65.8)	107 (58.5)	
2,000–4,999	122 (31.9)	66 (36.1)	0.071
≥5,000	9 (2.3)	10 (5.5)	
Clinical data			
Disease duration (years)	6.48 (6.46)	6.73 (5.80)	0.647
Familial history (%)	48 (12.5)	25 (13.7)	0.690
Side effects (%)	130 (33.9)	62 (33.9)	1.000
Poor adherent (%)	15 (3.9)	15 (8.2)	0.044*
Malignancy (%)	32 (8.4)	18 (9.8)	0.635
Operation history (%)	61 (15.9)	22 (12.0)	0.254
Fracture history (%)	64 (16.7)	25 (13.7)	0.389
Number of combined disorders	0.90 (1.11)	0.84 (0.92)	0.542
Physician's VAS score (mm)	19.46 (12.65)	17.21 (14.15)	0.058
Pain VAS score (mm)	22.35 (21.44)	22.15 (21.49)	0.918
Global health VAS score (mm)	24.56 (19.43)	25.30 (20.91)	0.680
Fatigue VAS score (mm)	34.54 (27.52)	36.38 (26.75)	0.454
DAS28-ESR	2.35±0.64	2.40±0.61	0.377
EQ-5D score	0.78±0.17	0.78±0.17	0.962
HAQ score	0.39±0.47	0.34±0.40	0.185
Laboratory data			
RF (%)	184 (48.0)	95 (51.9)	0.419
ACPA (%)	246 (64.2)	126 (68.9)	0.299
Hemoglobin (g/dL)	12.79±1.20	12.92±1.40	0.230
ESR (mm/h)	14.89±11.92	16.14±12.98	0.253
CRP (mg/dL)	0.31±0.62	0.32±0.64	0.750
Medication data			
Methotrexate (%)	327 (85.4)	165 (90.2)	0.142
Number of DMARDs	1.78±0.65	1.77±0.64	0.789
NSAIDs (%)	304 (79.4)	127 (69.4)	0.011*
Steroids (%)	262 (68.4)	120 (65.6)	0.503
Biologics (%)	17 (4.4)	11 (6.0)	0.414

ACPA, anti-citrullinated protein antibody; BMI, body mass index; CRP, C-reactive protein; DAS28, 28-joint count disease activity score; DMARD, disease modifying anti-rheumatic drug; ESR, erythrocyte sedimentation rate; EQ-5D, EuroQol-5D; HAQ, health assessment questionnaire; NSAID, non-steroidal anti-inflammatory drug; RF, rheumatoid factor; VAS, visual analogue scale; *$p < 0.05$.

Supplementary Table 2. Cox proportional hazards model of flares in patients with RA who exhibit remission

Variables	Univariate analysis		Multivariate analysis	
	HR (95% CI)	*p*-value	HR (95% CI)	*p*-value
Monthly income (USD)				
<2,000	1 (reference)		1 (reference)	
2,000-4,999	0.94 (0.74-1.18)	0.575	1.03 (0.80–1.31)	0.833
≥5,000	0.54 (0.31-0.95)	0.033*	0.62 (0.35–1.09)	0.099
Physician's VAS score	1.02 (1.02-1.03)	0.000**	1.02 (1.01–1.03)	0.000**
Hemoglobin	0.90 (0.83-0.98)	0.021*	0.90 (0.83–0.98)	0.028*
CRP	1.39 (1.05-1.85))	0.024*	1.06 (0.74–1.52)	0.744
EQ–5D score	0.42 (0.22-0.80)	0.008**	0.64 (0.30–1.37)	0.248
HAQ score	1.22 (1.04-1.44)	0.016*	1.28 (1.08–1.52)	0.006**

CRP, C-reactive protein, EQ-5D, EuroQol-5D; HAQ, health assessment questionnaire; HR, hazard ratio; VAS, visual analogue scale; *$p < 0.05$; **$p < 0.01$.
